# Perineural or intravenous administration of dexmedetomidine combined with anterior quadratus lumborum block for analgesia after retroperitoneal laparoscopic surgery: a prospective, randomized controlled trial

**DOI:** 10.1186/s12871-026-04103-4

**Published:** 2026-07-18

**Authors:** Yunru Jiang, Hengrui Zhang, Chenyang Shi, Yang Niu, Han Gao, Yuxiang Meng, Zhibiao Xu, Li Zhang, Sumin Yuan, Zijie Ling, Linlin Zhao

**Affiliations:** 1 Jiangsu Province Key Laboratory of Anesthesiology, Xuzhou Medical University, Xuzhou, China; 2 Department of Anesthesiology, The Affiliated Hospital of Xuzhou Medical University, NO. 99 Huaihai Road, Quanshan District, Xuzhou City, 221002 China; 3 Department of Anesthesiology, Kunshan Women and Children’s Healthcare Hospital, Suzhou, China; 4 Department of Anesthesiology, The Second Affiliated Hospital of Xuzhou Medical University, Xuzhou, China; 5 Department of Anesthesiology, The First People’s Hospital of Changzhou, Changzhou, China; 6 Department of Anesthesiology, The People’s Hospital of Wujin, Changzhou, China

**Keywords:** Dexmedetomidine, Quadratus lumborum block, Retroperitoneal laparoscopic surgery, Postoperative analgesia

## Abstract

**Purpose:**

The duration of a single anterior quadratus lumborum block (QLB) cannot fully meet the postoperative analgesic needs of patients undergoing retroperitoneal laparoscopic surgery. We investigated whether perineural or intravenous dexmedetomidine (Dex) administration combined with anterior QLB could improve analgesic efficacy.

**Methods:**

Ninety-six patients were randomized, and 88 were included in the modified per-protocol analysis. A total of 88 patients scheduled for retroperitoneal laparoscopic surgery under general anesthesia combined with anterior QLB were randomly assigned to one of three groups: (1) control group (R): 0.375% ropivacaine 30 mL; (2) ropivacaine plus perineural Dex (RD): 0.375% ropivacaine 30 mL plus 1 µg/kg Dex, (3) ropivacaine plus intravenous Dex (RDiv): 0.375% ropivacaine 30 mL plus intravenous 1 µg/kg Dex. The primary outcome was the 48 h postoperative sufentanil consumption. Secondary outcomes included Numerical Rating Scale (NRS) scores at different postoperative time points, intraoperative hemodynamic parameters, and adverse effects.

**Results:**

The 48 h postoperative sufentanil consumption was significantly lower in group RD than in groups R and RDiv [median (IQR): 31.0 (16.3, 39.0) vs. 40.1 (27.2, 60.7) vs. 42.0 (31.5, 57.9); adjusted *P* < 0.05, Bonferroni post-hoc test], with between-group differences of -17.2 µg (95%CI -23.4 to -11.3) and − 16.7 µg (95%CI -23.2 to -10.8). Fewer patients in group RD (14% vs. 40% vs. 34%; *P* = 0.069) required rescue analgesia, though the difference did not reach statistical significance. Group RD exhibited significantly lower resting and dynamic NRS pain scores at key postoperative time points (*P* < 0.05). QoR-15 was statistically higher in group RD, although the between-group difference was below the reported minimal clinically important difference (MCID). Dexmedetomidine was associated with lower MAP and HR at selected intraoperative time points, without a statistically significant increase in postoperative bradycardia or hypotension. No significant differences were observed among the three groups in adverse events, time to first flatus, ambulation, or length of stay( all *P* > 0.05).

**Conclusions:**

Perineural administration of 1 µg/kg dexmedetomidine combined with anterior QLB could provide better postoperative analgesia, which reduced 48-hour postoperative sufentanil consumption and improved pain scores at several time points. However, intravenous dexmedetomidine at the same dose did not confer comparable benefits in this setting.

**Trial registration:**

Chinese Clinical Trial Registry (ChiCTR2300074778) Registration date 16/08/2023.

**Supplementary Information:**

The online version contains supplementary material available at https://doi.org/10.1186/s12871-026-04103-4.

## Introduction

As a branch of laparoscopic surgery, retroperitoneal laparoscopic surgery has been widely used in various urological surgeries. Laparoscopic surgery can significantly reduce postoperative incision pain compared to open abdominal surgery, but postoperative pain caused by various factors such as surgical manipulation, CO_2_ pneumoperitoneum, postoperative gastrointestinal symptoms, and stimulation of abdominal nerves is still unavoidable. Persistent pain not only impairs patients’ daily activities but also affects their psychological and physical status, thereby reducing the quality of postoperative recovery and prolonging hospital stay [1].

Quadratus lumborum block (QLB) is a fascial plane block that relieves somatic and visceral pain by spreading local anesthetics to the paravertebral space. It serves as a supplement to general anesthesia for perioperative analgesia in retroperitoneal laparoscopic surgery [2–4]. However, the analgesic duration of a single anterior QLB is limited by the pharmacokinetic properties of local anesthetics, which often fails to cover the entire postoperative pain period.

Dexmedetomidine (Dex), a highly selective α_2_-adrenoceptor agonist, has been widely used as a local anesthetic adjuvant in peripheral nerve blocks. It exerts sedative, analgesic, and anti-inflammatory effects [5], and previous studies have demonstrated that it can significantly prolong analgesic duration and reduce the opioid consumption without major adverse events [6, 7].

However, the optimal administration route of dexmedetomidine as an adjuvant to anterior QLB remains unclear, and high-quality evidence is lacking. This prospective, double-blind, randomized controlled trial was designed to test the hypothesis that perineural dexmedetomidine combined with anterior QLB provides better postoperative analgesia than intravenous administration in patients undergoing retroperitoneal laparoscopic surgery.

The primary objective of this study was to compare the effects of perineural versus intravenous dexmedetomidine combined with anterior QLB on postoperative 48-hour sufentanil consumption in patients undergoing retroperitoneal laparoscopic surgery [8]. Secondary objectives included assessing pain scores at different postoperative time points, intraoperative hemodynamic stability, incidence of adverse events, and patient recovery quality.

## Materials and methods

### Study design

This prospective, double-blind, randomized controlled trial was conducted from August to December 2023 at the Affiliated Hospital of Xuzhou Medical University in accordance with the Declaration of Helsinki. The study was approved by the Ethics Committee of The Affiliated Hospital of Xuzhou Medical University (XYFY2023-KL150-02) and was registered at the Chinese Clinical Trial Registry (ChiCTR2300074778; registration date: 16/08/2023). Perineural dexmedetomidine was used off-label in this trial. This off-label administration was approved by the institutional ethics committee and fully disclosed in the written informed consent provided to all participants. The date of first patient enrollment was 20/08/2023. Written informed consent was obtained from all patients before enrollment. This study was designed and reported in accordance with the Consolidated Standards of Reporting Trials (CONSORT) guidelines.

### Participants

A total of 96 patients aged 18–75 years with American Society of Anesthesiologists physical status classification (ASA) grade Ⅰ to III and BMI 18.5 to 30.0 kg/m^2^ who were scheduled for elective retroperitoneal laparoscopic surgery were included. Exclusion criteria included: contraindications to regional nerve block, such as local infection at the puncture site or severe coagulation disorders; allergies to any of the drugs used in this trial; severe cardiovascular, cerebrovascular, respiratory, hepatic, or renal disease; diabetes; history of chronic pain or long-term use of opioids, steroids, or NSAIDs; inability to understand or complete study assessments; pre-existing bradycardia (HR < 50 bpm); or participation in another clinical trial.

### Randomization and blinding

Consenting study participants were randomized using a computer-generated random number list with simple randomization (1:1:1 allocation ratio). No permuted blocks or stratification factors were used. The allocation sequence was sealed in sequentially numbered opaque envelopes and stored by an independent research coordinator. On the day of surgery, the research coordinator delivered one envelope per patient to the anesthesia assistant, who prepared all the study solutions and remained uninvolved in subsequent clinical procedures. Patients, anesthesiologists performing the QLB, outcome assessors, and statisticians were all blinded to the group allocation.

### Group processing

All study patients received study solutions according to their group allocation as follows:

1. Group R: 30 mL of 0.375% ropivacaine + 1 mL normal saline for perineural injection; 50 mL normal saline administered intravenously (i.v.).
2. Group RD: 30 mL of 0.375% ropivacaine containing 1 µg/kg Dex for perineural injection; 50 mL normal saline administered i.v.
3. Group RDiv: 30 mL of 0.375% ropivacaine + 1 mL normal saline for perineural injection; 1 µg/kg Dex administered i.v. in 50 mL normal saline.

All intravenous solutions were infused at a rate of 200 mL/h 15 min before the start of the operation. The independent anesthesia assistant prepared all study solutions off-site. Dexmedetomidine was pre-diluted with normal saline before mixing with ropivacaine to guarantee identical visual appearance (colour, transparency and turbidity) of all perineural solutions.

### Anesthesia procedure

All patients fasted from food and water routinely before surgery. Upon the arrival of patients at the operating room, routine data such as electrocardiography (ECG), heart rate (HR), invasive arterial blood pressure (IBP) and peripheral oxygen saturation (SpO_2_) were monitored, and the intravenous access was established on the patient’s nonoperative side. The patient then inhaled oxygen by mask at 5 L/min. Before block performance, midazolam (0.02 mg/kg) and sufentanil (0.08 µg/kg) were intravenously injected for anxiolysis and analgesia as needed.

Patients were positioned in the lateral decubitus position with the operative side facing up. The region for nerve block anesthesia on the operative side was prepared in a sterile manner and covered with a disposable surgical drape. The ultrasound machine (NAVIS, Shenzhen Wisonic Medical Technology Co., Ltd) was used for localization, which could help improve the accuracy and safety of block puncture and reduce the risk of complications. The low frequency convex array probe was placed on the midaxillary line between the costal margin and the iliac crest. With ultrasound, the transverse processes could be seen, and the quadratus lumborum muscle could be found connected to the tip of the transverse process. The surrounding structures, such as the psoas major muscle, erector spinae muscles and kidney, could also be identified.

Local infiltration anesthesia was performed with 1% lidocaine. Following the insertion of the needle posterior to the ultrasound transducer in an oblique anterior approach, the disposable nerve stimulation needle (Stimuplex D Plus, B. Braun, Melsungen, Germany) passed through the quadratus lumborum muscle and was inserted into the middle layer of the thoracolumbar fascia between the quadratus lumborum and psoas major muscles. After a negative aspiration test for blood in the thoracolumbar fascial interspace, 30 mL of the mixture prepared beforehand was injected. The spread of the local anesthetic in the thoracolumbar fascia could be visualized by ultrasound. The block efficacy was evaluated via cold sensation testing. A satisfactory nerve block effect could be achieved when obvious hypoesthesia occurred in the anterior and lateral abdominal wall between the ribs and the inguinal region. The procedure was defined as a failure when there was an absence of hypoesthesia 30 min later. All QLB procedures were performed by an experienced chief anesthesiologist.

The anesthesia induction started when the effects of QLB occurred. After the inhalation of pure oxygen for 5 min, the patient was given an intravenous infusion of midazolam 0.05 mg/kg, etomidate 0.3 mg/kg, sufentanil 0.5 µg/kg and cisatracurium 0.2 mg/kg for anesthesia induction and mechanical ventilation after insertion of a laryngeal mask (Ambu AuraGain, Ambu A/S, Denmark). The volume control mode with a tidal volume of 6–8 mL/kg and a ventilation frequency of 12–18 times/min was adopted, and the P_ET_CO_2_ was maintained at 35–45 mmHg. No patients required conversion to tracheal intubation in this study.

Anesthesia maintenance: Intravenous infusion of propofol 2–6 mg·kg^− 1^·h^− 1^, remifentanil 0.1–0.3 µg·kg^− 1^·min^− 1^, inhalation of 1% sevoflurane, and intermittent administration of cisatracurium 0.03 mg/kg as needed. During the operation, mean arterial pressure (MAP) was maintained at the baseline value ± 20%, heart rate (HR) 50–99 bpm, and the BIS value 40–60. If MAP decreased by more than 20% of the baseline value, the patient should be given 20–40 µg of phenylephrine and, if necessary, the drug should be administered again. Alternatively, the patient should be treated with rapid intravenous fluids of the appropriate type and volume as judged by the anesthesiologist. If MAP increased by more than 20% of the baseline value, the infusion rate of propofol and remifentanil should be adjusted. When HR was less than 50 bpm, atropine 0.2–0.5 mg was administered, repeating if necessary. Ketorolac tromethamine 30 mg was injected 20 min before the end of the surgery. All anesthetics were discontinued upon completion of wound closure.

Patients were transferred to the post-anesthesia care unit (PACU) after surgery, and the laryngeal mask was removed when the extubation indications were met. All patients received patient-controlled intravenous analgesia (PCIA) after surgery. Sufentanil 2 µg/kg and tropisetron 6 mg were added to normal saline and diluted to 100 mL, with no background infusion, 2 mL per press, and a 15-min lockout interval. Ketorolac tromethamine 30 mg would be injected as rescue analgesia if numerical rating scale (NRS) scores were still greater than 4 after PCIA or any additional analgesic was required.

### Outcome measurements

The primary outcome was the 48 h postoperative sufentanil consumption. Secondary outcomes included the intraoperative consumption of propofol and remifentanil; mean arterial pressure (MAP) and heart rate (HR) measured at six time points: before quadratus lumborum block (T0), at lateral decubitus position (T1), at skin incision (T2), 30 min after skin incision (T3), at wound closure (T4), and 30 min after laryngeal mask removal (T5); the number of patients requiring rescue analgesia; resting numerical rating scale (NRS) scores (0 = no pain, 10 = worst pain imaginable) [9] at 2, 6, 12, 24, and 48 h postoperatively, as well as dynamic NRS scores (assessed when patients turned over independently) at the same time points. We also monitored postoperative adverse events using consistent definitions: bradycardia was sustained heart rate < 50 bpm for ≥ 1 min, hypotension was MAP falling below 80% of baseline for ≥ 5 min, and nausea and vomiting were counted for any episode within 72 h postoperatively. At 2 h postoperatively, sedation depth was scored via the 1–6 Ramsay sedation scale [10]: 1 = agitated, 2 = calm and cooperative, 3 = drowsy and follows commands, 4 = asleep and arousable, 5 = asleep and difficult to arouse, 6 = deeply asleep and unarousable. Lower limb muscle weakness was defined as a Medical Research Council (MRC) grade [11] < 5 at 2 h postoperatively: 0 = no muscle contraction, 1 = slight muscle contraction, 2 = limb movement in the horizontal plane, 3 = limb elevation against gravity, 4 = limb movement against partial resistance, 5 = normal muscle strength. Recovery endpoints included time to first flatus, time to first ambulation, postoperative hospital length of stay, and the 48-hour postoperative 15-item Quality of Recovery (QoR-15) score [12].

### Sample size calculation

The sample size was calculated using PASS (Power Analysis and Sample Size) 15.0 software. Based on a pilot study of five patients in each group, the 48 h postoperative sufentanil consumption was (46.7 ± 25.5), (25.4 ± 22.0) and (39.5 ± 27.3) in group R, group RD and group RDiv respectively. Assuming a statistical power of 0.80 and a type I error of 0.05 using PASS 15.0, with a dropout rate of 15%, the required sample size was ultimately 32 per group.

### Statistical analysis

SPSS 26.0 software was used for data analysis. The Shapiro-Wilk test was used to assess the normality of data distribution. Numerical variables with a normal distribution were presented as mean ± standard deviation (SD) and analyzed using one-way analysis of variance (ANOVA) with post hoc tests. Non-normally distributed continuous variables were presented as median and interquartile range (IQR) and were analyzed using the Kruskal-Wallis H test and pairwise multiple comparisons. Categorical variables were expressed as frequency (n) or percentage (%) and were analyzed by chi-square test *(χ2)* test or Fisher’s exact test. *P* < 0.05 indicated the difference was statistically significant. The Bonferroni correction was applied for multiple comparisons to maintain an overall type I error rate of 0.05. The primary analysis was performed according to a modified per-protocol principle. In addition, an intention-to-treat (ITT) sensitivity analysis including all 96 randomized participants was performed to verify result robustness. Missing sufentanil data of three withdrawn patients were imputed via last-observation-carried-forward (LOCF). Two block-failure patients and two ICU-transfer patients remained in their original allocated groups and were regarded as treatment failures in ITT analysis.

## Results

**Fig. 1:**
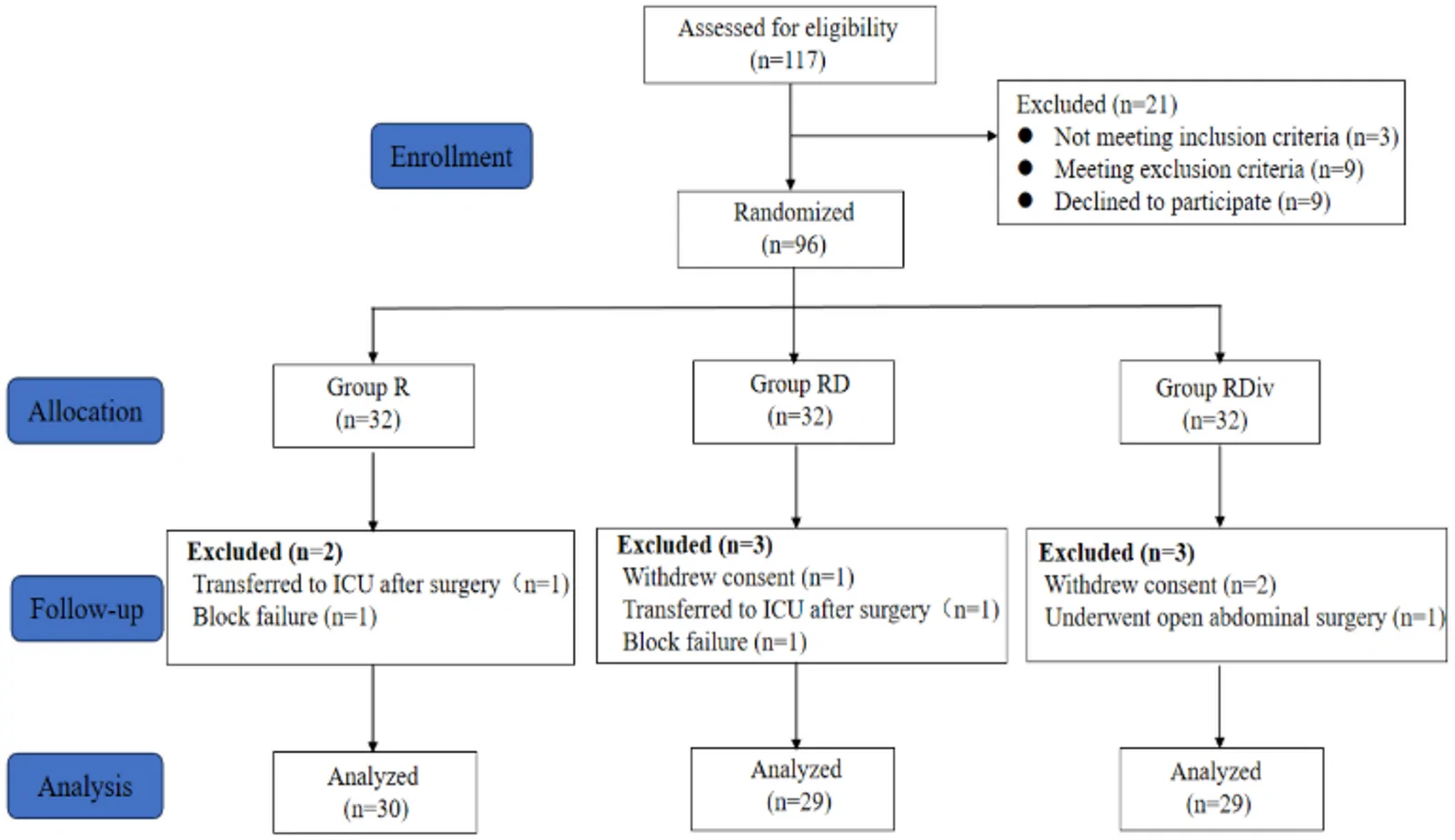
CONSORT flowchart of the study

A total of 117 patients were assessed for eligibility, of whom 21 were excluded before randomization, leaving 96 patients randomized (32 per group) and included in the ITT dataset. Eight patients with post-randomization protocol deviations were excluded from the primary modified per-protocol analysis: one conversion to open surgery, two block failures, two ICU transfers and three withdrawals. Ultimately, 30 patients in group R, 29 in RD and 29 in RDiv entered the primary analysis (Fig. 1).

There were no significant differences among the three groups in terms of gender, age, BMI, ASA grade, hypertension (diagnosed history), side of surgery. The duration of surgery (min), type of surgery and intraoperative medication were also similar across groups (Table 1).

**Table 1:** Patient characteristics

	Group R (*n* = 30)	Group RD (*n* = 29)	Group RDiv (*n* = 29)	*P*-value
Gender (Male / Female)	18/12	19/10	18/11	0.907
Age (years)	53.8 ± 11.7	51.3 ± 12.3	49.4 ± 12.9	0.398
BMI (kg/m^2^)	25.1 ± 2.8	25.4 ± 3.0	24.6 ± 3.0	0.582
ASA (Ⅰ / Ⅱ / Ⅲ)	6/22/2	7/20/2	10/17/2	0.758
Hypertension	12 (40%)	14 (48%)	7 (24%)	0.155
Side of surgery (Left / Right)	13/17	21/8	15/14	0.070
Type of surgery				0.788
Partial nephrectomy	5 (17%)	3 (10%)	3 (10%)	
Radical nephrectomy	6 (20%)	3 (10%)	6 (21%)	
Renal cyst decompression	11 (37%)	11 (37%)	7 (24%)	
Partial adrenalectomy	5 (17%)	8 (28%)	7 (24%)	
Ureterolithotomy	3 (10%)	4 (14%)	6 (21%)	
Duration of surgery (min)	73 [59,121]	90 [60,115]	90 [60,120]	0.401
Propofol consumption (mg)	200 [170,350]	250 [175,310]	260 [210,350]	0.250
Remifentanil consumption (µg)	800 [700,1500]	850 [625,950]	900 [690,1350]	0.348

Values are presented as mean ± SD or number (%) or median [IQR]

*Abbreviations*: *BMI* Body mass index, *ASA* American Society of Anesthesiologists

**Fig. 2:**
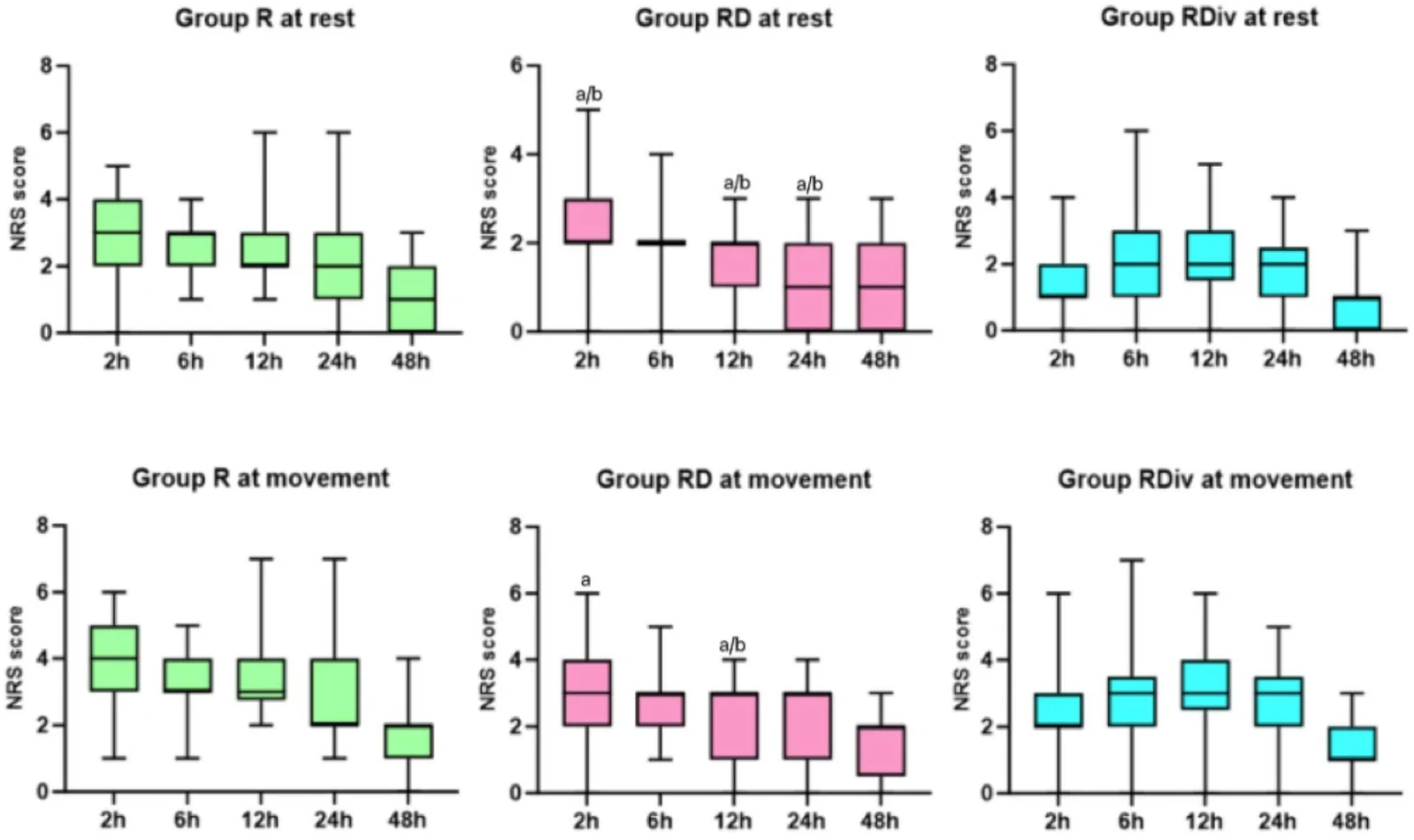
Box plot of NRS scores during rest and movement. After Bonferroni adjustment, **a**: Group RD vs. Group R, *P*<0.05; **b**: Group RD vs. Group RDiv, *P*<0.05

The 48 h postoperative sufentanil consumption was significantly lower in group RD than in groups R and RDiv [median (IQR): 31.0 (16.3, 39.0) vs. 40.1 (27.2, 60.7) vs. 42.0 (31.5, 57.9); adjusted *P* < 0.05, Bonferroni post-hoc test; Table 2]. Hodges-Lehmann estimation showed that sufentanil consumption was significantly lower in group RD than in group R (difference − 17.2 µg, 95%CI -23.4 to -11.3, *P* = 0.007) and group RDiv (difference − 16.7 µg, 95%CI -23.2 to -10.8, *P* = 0.005). Fewer patients in group RD required rescue analgesia than in group R (14% vs. 40%; absolute risk reduction 26% [95% CI 6% to 43%]; number needed to treat = 4 [95% CI 3 to 17]; *P* = 0.039). However, after Bonferroni correction for multiple comparisons, the difference was not statistically significant. Resting NRS scores at 12 and 24 h and dynamic NRS scores at 12 h were significantly lower in Group RD postoperatively (all *P* < 0.05; Fig. 2). At 2 h postoperatively, both group RD and group RDiv had lower scores than group R (*P* < 0.05), with no difference between the two Dex groups.

**Table 2:** Postoperative analgesic consumption and pain scores

	Group *R* (*n* = 30)	Group RD (*n* = 29)	Group RDiv (*n* = 29)	*P*-value
NRS scores at rest				
2 h	3.0 ± 1.4	2.2 ± 1.0^a, b^	1.5 ± 1.1^a^	< 0.001
6 h	2.6 ± 0.9	2.0 ± 0.9	2.0 ± 1.4	0.042
12 h	2.6 ± 1.3	1.7 ± 1.0^a, b^	2.3 ± 1.2	0.015
24 h	2.2 ± 1.4	1.3 ± 1.1^a, b^	1.9 ± 1.0	0.012
48 h	1.1 ± 0.8	1.0 ± 0.9	0.9 ± 0.8	0.745
NRS scores at movement				
2 h	3.9 ± 1.2	3.0 ± 1.2^a^	2.4 ± 1.4^a^	< 0.001
6 h	3.3 ± 0.8	2.7 ± 1.1	2.8 ± 1.7	0.112
12 h	3.4 ± 1.3	2.3 ± 1.3^a, b^	3.1 ± 1.3	0.008
24 h	2.8 ± 1.4	2.2 ± 1.2	2.7 ± 1.3	0.144
48 h	1.8 ± 0.9	1.5 ± 1.1	1.4 ± 0.9	0.238
Postoperative sufentanil consumption (µg)	40.1[27.2, 60.7]	31.0[16.3, 39.0]^a, b^	42.0[31.5, 57.9]	0.006
Number of patients with rescue analgesia (%)	12(40%)	4 (14%)	10 (34%)	0.069

Values are presented as mean ± SD or numbers (%) or median [IQR]

*Abbreviation*: *NRS* Numerical rating scale

Statistical significance was defined as *P* < 0.05 after Bonferroni correction; ^a^*P* < 0.05 versus group R; ^b^*P* < 0.05 versus group RDiv

**Fig. 3:**
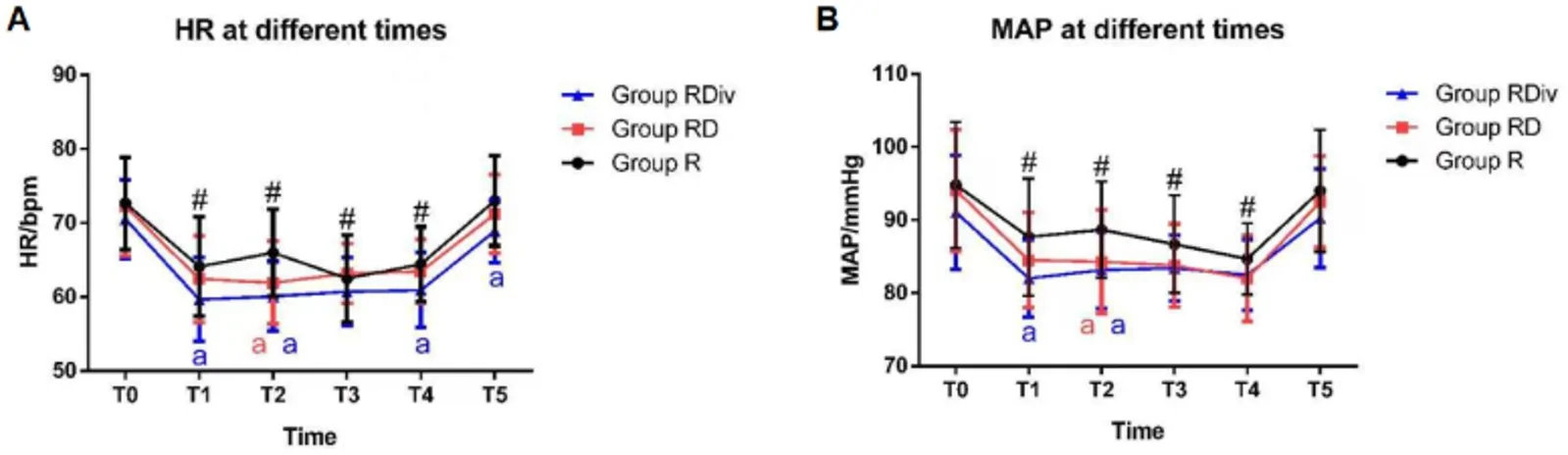
Intraoperative hemodynamic data. **A** Heart rate. **B** Mean arterial pressure. Values are expressed as mean ± standard deviation. ^a^*P* < 0.05 versus group R; ^#^*P* < 0.05 versus T0.(T0: before quadratus lumborum block; T1: at lateral decubitus position; T2: at skin incision; T3: 30 min after skin incision; T4: at wound closure; T5: 30 min after laryngeal mask removal)

MAP and HR at T2 in group RD were significantly lower than those in group R (adjusted *P* < 0.05). MAP at T1, T2 and HR at T1, T2, T4, T5 in group RDiv were significantly lower than those in group R (*P* < 0.05). Compared with MAP and HR at T0, the two items at T1, T2, T3, T4 were significantly lower within each group (*P* < 0.05; Fig. 3).

A sensitivity analysis was conducted for the primary outcome (48-hour sufentanil consumption) using rank-based ANCOVA stratified by side of surgery (left/right). After adjusting for the potential imbalance in surgical laterality, group RD still exhibited significantly lower sufentanil consumption compared with group R and group RDiv (both *P* < 0.05). The overall conclusion remained unchanged, confirming that the marginal imbalance in side of surgery did not affect the primary outcome. The ITT sensitivity analysis of all 96 randomized patients yielded consistent results with the primary mPP analysis, with RD group showing significantly lower postoperative sufentanil consumption (Supplementary Table S1).

The time to first flatus and ambulation after surgery and length of stay after surgery were statistically comparable among the three groups (all *P* > 0.05). The 48-hour QoR-15 score in Group RD was significantly higher than that in group R and group RDiv (*P* < 0.05). No significant differences were observed among the three groups in postoperative bradycardia, hypotension, nausea and vomiting, and lower limb weakness (all *P* > 0.05; Table 3).

**Table 3:** Postoperative recovery and adverse reactions

	Group R (*n* = 30)	Group RD (*n* = 29)	Group RDiv (*n* = 29)	*P*-value
Time to first flatus (h)	25.50 [20.00, 31.50]	23.00 [18.75, 32.00]	24.00 [20.25, 28.75]	0.654
Time to first ambulation (h)	23.50 [22.00, 27.50]	23.00 [17.75, 28.00]	26.00 [21.00, 30.00]	0.371
Length of stay after surgery (days)	5.00 [3.25, 6.00]	4.00 [3.00, 5.50]	4.00 [3.00, 6.00]	0.254
48 h QoR-15 score	124.3 ± 5.3	128.5 ± 7.1^a^	126.5 ± 5.3	0.030
Postoperative hypotension (%)	2 (7%)	1 (3%)	2 (7%)	1.000
Postoperative bradycardia (%)	0 (0%)	1 (3%)	2 (7%)	0.318
Nausea and vomiting (%)	10 (33%)	6 (21%)	7 (24%)	0.519
Ramsay sedation scores	2 [2, 2]	2 [2, 2]	2 [2, 2]	1.000
Lower limb weakness (%)	4 (13%)	4 (14%)	5 (17%)	0.659

Values are presented as mean ± SD, numbers(%) or median [IQR]

*Abbreviation*: *QoR-15* Quality of Recovery-15

Statistical significance was defined as *P* < 0.05 after Bonferroni correction; ^a^*P* < 0.05 versus group R; ^b^*P* < 0.05 versus group RDiv

## Discussion

In this study, we found that perineural administration of Dex (1 µg/kg) combined with anterior QLB significantly improved postoperative analgesia in terms of less sufentanil consumption and better pain scores. Additionally, perineural Dex led to mild reductions in intraoperative MAP and HR at partial surgical time points and higher 48-hour QoR-15 score, without a statistically significant increase in adverse events. In contrast, intravenous infusion of 1 µg/kg Dex provided no clear benefit.

The QLB is a widely used truncal fascial plane block with multiple approaches, including lateral, posterior, anterior, and intramuscular techniques [13]. The anterior QLB, providing sensory coverage from T4 to L1, shows a favorable effect on urological surgery [14, 15]. However, the analgesic duration of a single QLB is limited by ropivacaine pharmacokinetics, typically ranging from several to over 20 h, which is insufficient to cover the full postoperative pain period following retroperitoneal laparoscopic surgery [16–18]. Consequently, adjunctive agents capable of prolonging block duration and improving analgesic quality have attracted considerable clinical interest.

Dex is a highly selective α_2_-adrenoceptor agonist, which has pharmacological characteristics including sedative, hypnotic, analgesic, anxiolytic, and anti-inflammatory effects [7]. The effects of dexmedetomidine on neural activity are mediated through the inhibition of neuronal potassium and sodium channels and subsequent blockade of the hyperpolarization-activated cation current [19]. Multiple clinical trials showed that Dex, when used as an adjuvant for nerve block, could accelerate the onset of the block, prolong the duration of analgesia and improve the quality of analgesia [20–22]. Previous studies have confirmed the safety and effectiveness of 1 µg/kg Dex mixed with local anesthetics, so we chose the same dose for our study [23, 24].

Whether intravenous Dex affects the postoperative analgesic efficacy of nerve block remains controversial [25]. Abdallah et al. reported that 0.5 µg/kg Dex administered perineurally and intravenously similarly improved the effect of analgesia after arthroscopic shoulder surgery [26]. However, Jin et al. reported that intravenous infusion of 0.5 µg/kg Dex had no enhancement of analgesic efficacy after femoral nerve block combined with sciatic nerve block [8]. Another study showed that intravenous infusion of 2 µg/kg Dex improved the pain after interscalene brachial plexus block and reduced the postoperative 24 h cumulative opioid consumption, but no similar effect was observed with 0.5 and 1.0 µg/kg Dex infused intravenously [27]. In our study, intravenous infusion of 1 µg/kg Dex did not significantly improve analgesic efficacy after anterior QLB, which was consistent with the result of the previous study by Jin et al. Differences in Dex dose, local anesthetic concentration and type, and surgical procedure might explain these discrepancies.

It is worth noting that the intravenous dexmedetomidine group (RDiv) received slightly more intraoperative remifentanil (median 900 µg) than the other two groups (800–850 µg). Prolonged remifentanil infusion may induce acute opioid tolerance [28], which could partly explain why the RDiv group did not show reduced postoperative sufentanil consumption. A strength of our study is that intraoperative opioid use was recorded and reported, allowing readers to consider this factor.

In the present study, the analgesic benefit appeared more pronounced with perineural administration than with intravenous administration. One possible explanation was that perineural dexmedetomidine may exert both peripheral and systemic effects, whereas intravenous administration primarily relied on central sympatholytic and sedative mechanisms [19]. Notably, local anesthetics such as ropivacaine may paradoxically induce neuroinflammation when administered perineurally. The local anti-inflammatory action of dexmedetomidine could therefore mitigate this adverse neural effect [29], potentially prolonging block duration and improving analgesia.

Postoperative pain scores were generally low across all groups, indicating that anterior QLB itself already provided satisfactory baseline analgesia [30]. Furthermore, clinically meaningful recovery outcomes, including time to first ambulation, time to first flatus, postoperative hospital stay, and rescue analgesic requirements, did not differ significantly among groups. The 48-hour QoR-15 score was higher in Group RD than in Group R (128.5 ± 7.1 vs. 124.3 ± 5.3, *P* = 0.030). However, the observed mean difference of 4.2 points was below the published minimal clinically important difference (MCID) of approximately 8 points for the QoR-15 scale in surgical patients [12, 31]. Consequently, although statistically significant, this difference may not represent a clinically meaningful improvement in the patients’ perception of recovery quality.

Hypotension and bradycardia are commonly observed after intravenous infusion of Dex, whereas the safety profile of perineural administration remains controversial [32]. Numerous studies have shown that the addition of Dex to local anesthetics had no effect on the occurrence of postoperative nausea and vomiting, bradycardia and hypotension [33], while Elyazed et al. found that 100 µg Dex could prolong the duration of sensory and motor block in patients undergoing subclavian brachial plexus block, and also increase the incidence of hypotension and bradycardia [34]. In this study, no increase in adverse reactions was found whether Dex was used as a local anesthetic adjuvant or was infused intravenously, which may be related to the small sample size. The study was powered specifically for the primary endpoint rather than for safety outcomes, and therefore may have been underpowered to detect differences in relatively infrequent adverse events. Consequently, the absence of statistically significant differences should not be interpreted as definitive evidence of safety equivalence.

There are some limitations to this study. First, the thoracolumbar fascia is a network of nerve fibers and blood vessels [35]. The concentration of Dex in the blood was not monitored in our study and we cannot rule out the systemic analgesic effect of Dex as an adjuvant for local anesthetics since systemic uptake can be influenced by site-specific perfusion levels [36]. Second, the time to first ambulation and length of stay after surgery were more likely to depend on the surgeon’s clinical instructions, limiting the ability to isolate anesthetic effects. Third, intravenous dexmedetomidine might produce recognizable physiological effects (e.g., sedation, bradycardia, hypotension) that could have compromised blinding of anesthesia providers and intraoperative personnel. We did not formally assess blinding integrity, and this potential functional unblinding should be considered when interpreting the results. Fourth, the study enrolled five different types of retroperitoneal laparoscopic procedures, which differed considerably in surgical invasiveness and expected postoperative pain. Randomization was not stratified by surgery type, and the sample sizes for adrenal and ureteral subgroups were too small to permit meaningful subgroup analyses. Fifth, midazolam and sufentanil were administered as needed before nerve block, and individual patient-level dosage records were not collected. Preoperative opioid exposure might alter postoperative sufentanil consumption, which represented an inherent confounding factor. Finally, this was a single-center study with a modest sample size, and the sample size calculation was based on a pilot study of only five patients per group. Consequently, the derived sample size carried uncertainty, and the post-hoc power might be lower than anticipated. Larger, more adequately powered trials are still needed for further investigation.

## Conclusions

In conclusion, perineural administration of 1 µg/kg dexmedetomidine as an adjuvant to anterior QLB provided superior postoperative analgesia by reducing 48-hour sufentanil consumption and improving pain control in patients undergoing retroperitoneal laparoscopic surgery. In comparison, intravenous dexmedetomidine at the same dose offered no comparable benefits. Our findings support the potential of perineural dexmedetomidine in this surgical population, but larger trials are needed to confirm its clinical value.

## Supplementary Information

Supplementary Material 1.

## Data Availability

The datasets generated and analyzed during the current study are available from the corresponding author on reasonable request. Individual participant data will not be shared publicly to protect patient confidentiality, but de‑identified data can be provided to qualified researchers after approval by the institutional ethics committee.
