# Supplementary material for: Perineural or intravenous administration of dexmedetomidine combined with anterior quadratus lumborum block for analgesia after retroperitoneal laparoscopic surgery: a prospective, randomized controlled trial
Source: BMC Anesthesiol. 2026 Jul 18;26:613. doi: 10.1186/s12871-026-04103-4 (PMC13660255; doi:10.1186/s12871-026-04103-4)
Supplement: Supplementary file 1 [file 12871_2026_4103_MOESM1_ESM.docx]

**Supplementary Table S1** ITT sensitivity analysis for the primary outcome.

|  | Group R (n=32) | Group RD (n=32) | Group RDiv (n=32) |
| --- | --- | --- | --- |
| 48-hour postoperative sufentanil consumption (μg) | 40.3 [27.5, 60.5] | 30.8 [16.0, 38.6] | 41.9 [31.2, 57.8] |

| Pairwise comparisons | Median difference (μg) | 95% CI | *P*-value |
| --- | --- | --- | --- |
| RD vs. R | -18.1 | -24.6 to -11.9 | 0.005 |
| RD vs. RDiv | -17.4 | -23.9 to -10.5 | 0.006 |
| R vs. RDiv | +1.2 | -5.3 to +7.8 | 0.726 |

Values are presented as mean ± SD or numbers(%) or median [IQR]. Statistical significance was defined as *P* < 0.05 after Bonferroni correction.
